# Feature Papers in Food Chemistry

**DOI:** 10.3390/molecules27248638

**Published:** 2022-12-07

**Authors:** Mirella Nardini

**Affiliations:** CREA, Research Centre for Food and Nutrition, Via Ardeatina 546, 00178 Rome, Italy; mirella.nardini@crea.gov.it

The Special Issue, entitled “Feature Papers in Food Chemistry”, is a collection of important high-quality papers (original research articles or comprehensive review papers) published in open access format. This Special Issue discusses new knowledge and new cutting-edge developments in the food chemistry field through selected works, in the hope of making a great contribution to the community through the dissemination of excellent research findings as well as by sharing innovative ideas in the field.

To be more specific, this Special Issue collects manuscripts on food composition with special emphasis on chemical characterization, the content of bioactive compounds, contaminants, and analytical aspects. The role of variety, cultivation, growing conditions, and technological processes on the nutritional quality of foods was also considered. Research studies dealing with the biological activity and healthy effects of food components were also presented.

The first research article of this Special Issue by Shi et al. [1] described the effect of different varieties and growing locations pertaining to the physicochemical properties of starch obtained from sweet potato root tuber. Three sweet potato varieties were studied: purple-, yellow-, and white-fleshed root tubers, planted in four growing locations. The starches isolated from root tubers were analyzed for size, crystalline structure, ordered degree, lamellar thickness, swelling power, water solubility and pasting, thermal and digestion properties, iodine absorption, and amylose content. Starches exhibited significantly different swelling powers, water solubility, pasting viscosities, thermal properties, amylose contents, iodine absorptions, and granule sizes. The ordered degree and lamellar thickness of starches were affected by different varieties and growing locations of sweet potatoes. Statistical analyses showed that starch physicochemical properties were significantly affected by different varieties and growing locations, as well as their interaction. Therefore, in consideration of starch applications, an appropriate variety for planting sweet potatoes in a specific growing location should be chosen.

The study of Markovska et al. [2] investigated the structural changes in β-casein as a function of temperature and pH, by means of nuclear magnetic resonance (NMR), as well as Fourier-transform infrared spectroscopy in conjunction with chemometric analysis. Both temperature (4 and 20 °C) and pH (3.9 and 7.0) strongly affected the secondary structure of the β-casein molecule, particularly the regions involving random coils and α-helix. The changes in secondary structure components were linked to the decreased hydrophobic interactions at lower temperatures and increasing pH levels. The results obtained may help to establish the structural behavior of β-casein during β-casein production, as well as process milk and milk-derived products. The combination of analytical methods used in this study can be further expanded to other proteins and assist in understanding protein behaviors during food processing.

Another study by He Z. [3] et al. comparatively evaluated the chemical composition and thermogravimetric behaviors of two types of cottonseed kernels: glanded and glandless cottonseed kernels. Chemical analysis and Fourier transform infrared (FTIR) spectroscopy showed that the two types of kernels had similar chemical components and mineral contents, but the former was slightly higher in protein, starch, and phosphorus content. The thermal behaviors and stability of the two types of cottonseed kernels were also investigated. The common glanded cottonseeds exhibited a high content of gossypol, a toxic compound. Glandless cottonseeds, in contrast, showed a trace gossypol level so that it can be used for animal feeds and human foods. The findings of this study enhance the potential application of glanded and glandless cottonseed kernels for food application.

The research article by He Y. [4] et al. investigated the efficacy of probiotics on constipation relief and their capacity to colonize the intestinal tract in mice. The probiotic compounds tested, including *Lactobacillus acidophilus*, *Lacticaseibacillus rhamnosus*, *Limosilactobacillus reuteri*, *Lactiplantibacillus plantarum*, and *Bifidobacterium animalis*, showed relief of constipation when administered to mice with loperamide-induced constipation. The effects of probiotics on constipation were associated with various aspects, including the gastrointestinal transit rate, the number and weight of stools, serum and gastrointestinal regulatory hormones, and serum cytokines. Moreover, some of the probiotic compounds were found to colonize the intestinal tract. This study yields a new perspective for the clinical use of probiotics to relieve constipation symptoms by combining strains with different mechanisms of action.

In the article of Malga et al. [5], the preservative properties of waste liquor obtained from octopus (*Octopus vulgaris*) cooking were investigated. Three different volumes of such liquor were included in the packaging medium employed for Atlantic Mackerel (*Scomber colias*) canning and were compared to control canned fish. The effects of octopus cooking liquor packaging on lipid hydrolysis, lipid oxidation, fatty acid profiles, and fatty acids ratios in canned mackerel were determined. The presence of the high and medium concentrations of octopus cooking liquor led to significantly lower levels of lipid oxidation and an average increase in the polyene index and monounsaturated fatty acid content with respect to control canned fish. This study constitutes a novel strategy to enhance the quality of commercial canned fish, thus enabling environmental sustainability and circular economy.

The study of Muchtaridi et al. [6] describes a new potential neuraminidase inhibitor from *Garcinia atroviridis* L. fruits and leaves. Neuraminidase is an enzyme preventing virion aggregation within the host cell and promoting cell-to-cell spread by cleaving glycosidic linkages to sialic acid, thus facilitating virus movement. The development of anti-influenza drugs that inhibit neuraminidase activity has emerged as a promising approach in the treatment of influenza. A bioassay-guided fractionation method was applied to isolate and identify the bioactive inhibitory compounds garcinia acid and naringenin from *Garcinia atroviridis* L. fruits and leaves, respectively. Garcinia acid demonstrated the highest inhibitory activity when compared to naringenin. Based on molecular docking results, garcinia acid was found to form strong ionic interaction with arginine triad residues of the viral neuraminidase, suggesting that garcinia acid has the potential to act as a neuraminidase inhibitor.

The article of Li Q. et al. [7] focused on the synthesis of four L-lysine-based gelators and their use to spur the formation of oleo-gels in four kinds of vegetable oils (corn germ oil, soybean oil, olive oil, and linseed oil). Gelation ability is not only affected by the structure of the gelators, but also by the composition of oils. The minimum gel concentration increased with the increase in the acyl-carbon chain length of the gelators. The strongest gelation ability was displayed in the olive oil for the same gelator. Rheological properties showed that the mechanical strength and thermal stability of the oleo-gels varied with the carbon-chain length of gelators and the type of vegetable oil.

In the study of Ognyanov et al. [8], the effects of gamma irradiation on dried rose hip (*Rosa canina* L.) phytochemicals were studied by employing several analytical techniques (GC-FID, HPLC-UV, HPSEC-RID, IR-FT, and SEM). The changes in macronutrients and micronutrients (e.g., carbohydrates, lipids, organic acids, and phenolics) were examined. The irradiation treatment increased the glucose content and released cellobiose, thus revealing cellulose destruction, and resulting in higher pectin yield. The exposure to the highest dose did not change the content of total carotenoids, β-carotene, ascorbic acid, and saturated and unsaturated fatty acids, while it affected tocopherols levels. A slight effect on conjugated dienes content was also measured with the highest dose. Gamma irradiation had a negligible effect on phenolic constituents (chlorogenic acid, quercetin, rutin, catechin, and epicatechin) and did not significantly affect antioxidant activity. From the results obtained, the authors concluded that the exposition of dried rose hip to gamma irradiation is safe and can be successfully applied for the microbial decontamination of fruits without affecting their nutritional value and biological activity.

The research article of Li Y. et al. [9] investigated the sizes, components, crystalline structures, and thermal properties of starches from 44 sweet potato varieties originating from 15 different countries, planted in the same growing conditions, to reveal the similarities and differences in their physicochemical properties. The granule size, iodine absorption, amylose content, X-ray diffraction patterns, and thermal properties of starches were studied, revealing significant differences among all sweet potato varieties. The thermograms of starches exhibited one-, two-, or three-peak curves, leading to a significantly different gelatinization temperature range. The significantly different starch properties divided the 44 sweet potato varieties into different groups due to their different genotype backgrounds. The research offers references for the utilization of sweet potato germplasm.

The study of Nagao et al. [10] investigated the effects of dried and fermented powders of edible algae (*Neopyropia Yezoensis*) on hepatic steatosis in obese mice. Edible algae *Neopyropia Yezoensis* was used as “Nori”, its dried product, in Japanese cuisine. In this study, Nori powder and Koji-fermented nori powder were used to supplement the diet of male *db/db* mice for 4 weeks. Eicosapentaenoic acid was found in the serum and liver of mice fed with nori or fermented nori diets in a dose-dependent manner. Both nori and fermented nori reduced hepatic triglyceride accumulation and hepatic injury with similar mechanisms. Although the eicosapentaenoic content of fermented Nori powder was one-third of that of nori powder, metabolomic analysis revealed that bioactive betaine analogs, such as stachydrine, betaine, and carnitine, were detected only in with fermented nori powder. In conclusion, the lipid components of nori, mainly eicosapentaenoic acid, are readily absorbable by the body to exert lipid-lowering effects. Moreover, the fermentation of nori produced anti-inflammatory and lipid-lowering betaine analogs, which, together with lipid components, can exert hepatic steatosis-alleviating effects.

The article by Cerulli et al. [11] provided a comprehensive analysis of green extracts of *Cynara cardunculus* subsp. *scolymus*, with cultivar “Carciofo di Paestum” PGI heads. Ethanolic/water extracts, infusions, and decoctions were analyzed by LC-ESI/QExactive/MS/MS and NMR analysis. A total of 17 compounds corresponding to caffeoylquinic acid derivatives, phenolics, flavonoids, and terpenoids were identified. Principal component analysis (PCA) showed significant differences among the extraction methods. Moreover, 5-caffeoylquinic acid (known as chlorogenic acid) and 1,5-dicaffeoylquinic acid (known as cynarin) quantization, phenolic content, antioxidant activity, and the α-glucosidase inhibitory activity of “Carciofo di Paestum” extracts were evaluated.

The second research article by Li Y. et al. [12] described the relationships between the X-ray diffraction peaks, molecular components, and heat properties of C-type starches from seven different sweet potato varieties. C-type starches contains both A- and B-type crystallinities. C-type starches with different proportions of A- and B-type crystallinities have different intensities and crystallinities of X-ray diffraction peaks. The relative crystallinities of diffraction peaks and total crystallinities showed some differences among some sweet potato varieties. These parameters were affected by amylose content, amylopectin structure, crystallinity type, and granule size. The thermograms of seven starches exhibited significant differences in peak shapes, widths, and gelatinization temperatures. The score plot of principle component analysis showed that the molecular components and heat properties parameters could differentiate the C-type starches and agreed with their characteristics of X-ray diffraction peaks. The starch properties, especially crystalline structure, affect the utilizations of starches. This study provides some information for the utilizations of C-type starches through their X-ray diffraction patterns.

In the research of Koulis et al. [13], the phenolic profile of Greek honey varieties was investigated using liquid chromatography–high-resolution mass spectrometry, and botanical origin markers were identified by metabolomic approaches. Three blossom honey markers were identified, namely galangin, pinocembrin, and chrysin, while gallic acid level was found to be significantly higher in oak honey. Twelve additional bioactive compounds were identified and semi-quantified, achieving comprehensive honey metabolomic characterization. Moreover, it was possible to discriminate thyme from blossom honey and develop discriminatory models with high predictive value.

The research article by Fontanella et al. [14] described a simplified method for the determination of herbicide glyphosate in white and brown rice by means of liquid chromatography coupled with inductively coupled mass triple quadrupole. This molecule, authorized for a wide variety of crop and non-crop uses, is an active substance in plant protection products. The debate on using glyphosate in Europe is still very active concerning the risk for human health. In 2015, the International Agency for Research on Cancer and the European Food Safety Agency put forward two conflicting views on the carcinogenic hazard of glyphosate to humans. In 2013, the European Commission established 0.1 mg/Kg as maximum residual level for rice, as the lower limit of analytical determination. Several methods are available for the determination of this molecule and its metabolites in different kinds of matrices, such as apples, cereals, soybean, as well as surface water and wastewater. The method proposed by Fontanella et al. is characterized by rapidity and simplicity in comparison with other existing procedures which are laborious and include several purification steps. In this study, the quantification of glyphosate was validated in terms of LOD, LOQ, accuracy, precision, linearity, and matrix effect. The matrix effect, which is a well-known critical problem in pesticide residue analysis, could be avoided by using a matrix-matched calibration line to avoid artefact results. The described procedure can be considered useful for the determination of glyphosate in different types of rice and designed and adapted for other cereals.

## Data Availability

Not applicable.

## Sample Availability

Samples of the compounds are not available from the authors.
